# Perioperative programmed death-1 expression on CD4^+ ^T cells predicts the incidence of postoperative infectious complications following gastrointestinal surgery

**DOI:** 10.1186/cc14111

**Published:** 2015-03-16

**Authors:** S Ono, T Ikeda, T Kubo, H Tsujimoto, M Kinoshita, T Ueno

**Affiliations:** 1 Hachioji Medical Center, Tokyo Medical University, Hachioji, Tokyo, Japan; 2 National Defense Medical College, Tokorozawa, Saitama, Japan

## Introduction

Programmed death-1 (PD-1) has been reported to be an immunoinhibitory receptor expressed by chronically stimulated T cells after T-cell activation. The present study was designed to evaluate the relationship between perioperative PD-1 expression on CD4^+ ^T cells and the incidence of postoperative infectious complications in patients undergoing gastroenterological surgery.

## Methods

This was a prospective observational study. The subjects of this study included 101 patients with gastroenterological disease who underwent elective abdominal surgery via laparotomy at the National Defense Medical College Hospital. Blood samples were taken on the preoperative day (Pre) and the first postoperative day (POD1). We calculated CD4^+ ^T-cell count and PD-1 expression on CD4^+ ^T cells by flow cytometer. The occurrence of postoperative infectious complications was defined according to a combination of clinical findings and the results of laboratory and other tests. The postoperative infectious complications in this study included incisional surgical site infections (SSIs), organ/space SSIs, enterocolitis, urinary tract infections, and pneumonia. Incisional and organ/space SSIs were diagnosed according to the definitions stated in the guidelines issued by the Center for Disease Control and Prevention.

## Results

Postoperative infectious complications occurred in 30 of the 101 patients. CD4^+ ^T-cell count was significantly lower in the patients who developed postoperative infectious complications at POD1 compared with those from the patients who did not. In addition, PD-1 expression on CD4^+ ^T cells was significantly higher at Pre or POD1 in patients who developed postoperative infectious complications. Those results were similar for the incidence of organ/space surgical site infection. Preoperative PD-1 expression on CD4^+ ^T cells tended to be higher in males than in females. We found there was a significant negative correlation between preoperative PD-1 expression on CD4^+ ^T cells and CD4^+ ^T-cell count.

## Conclusion

Perioperative CD4^+ ^T-cell count or PD-1 expression on CD4^+ ^T cells could be an early predictive marker for the development of postoperative infectious complications.

